# A testing cascade to identify repurposed insecticides for next-generation vector control tools: screening a panel of chemistries with novel modes of action against a malaria vector

**DOI:** 10.12688/gatesopenres.12957.2

**Published:** 2019-07-10

**Authors:** Rosemary Lees, Giorgio Praulins, Rachel Davies, Faye Brown, George Parsons, Anthony White, Hilary Ranson, Graham Small, David Malone

**Affiliations:** 1 Liverpool Insect Testing Establishment (LITE), Liverpool School of Tropical Medicine, Liverpool, L3 5QA, UK; 2 Vector Biology Department, Liverpool School of Tropical Medicine, Liverpool, L3 5QA, UK; 3 Innovative Vector Control Consortium (IVCC), Liverpool School of Tropical Medicine, Liverpool, Merseyside, L3 5QA, UK

**Keywords:** Vector control, Insecticide, Long lasting insecticidal nets, Indoor Residual Sprays, Anopheles gambiae, Resistance Management

## Abstract

**Background:** With insecticide resistance in malaria vectors spreading in geographical range and intensity, there is a need for compounds with novel modes of action to maintain the successes achieved to date by long-lasting insecticidal nets and indoor residual sprays, used as part of an insecticide resistance management strategy. Screening existing registered pesticides, predominantly those developed for use in agriculture, may provide a more rapid and less logistically challenging route to identifying active ingredients of value to public health than screening and chemical synthesis programmes for novel compounds.

**Methods:** Insecticides and acaricides from all IRAC classes, including those with unclassified modes of action, were assessed for inclusion in a laboratory bioassay testing cascade against adult female *Anopheles gambiae* mosquitoes. A longlist of representative candidate compounds was selected, excluding those with safety concerns, unsuitable physiochemical properties, and likely hurdles to registration for public health use.  An initial screen using topical application eliminated compounds with insufficient intrinsic activity, and a tarsal contact assay identified those with activity at an appropriate concentration. Compounds of interest were ranked by relative potency using dose response assays and discriminating dose calculations.

**Results:** Inclusion of an adjuvant enhanced the tarsal efficacy of several compounds, facilitating the promotion of chemistries with great potential, given suitable formulation, which would not progress based on activity of compound alone. Comparison of data between stages in the testing cascade suggest that a more streamlined approach, topical application to test for intrinsic activity and determining the discriminating dose to compare relative potency of compounds, may be sufficient to identify compounds with potential value for use in long lasting insecticidal nets and indoor residual spray products.

**Conclusions:** Identified were 11 compounds of interest as vector control agents (in descending order of potency): clothianidin, spinetoram, metaflumizone, dinotefuran, indoxacarb, abamectin, sulfoxaflor, oxazosulfyl, triflumezopyrim, fenpyroximate, and tolfenpyrad.

## Introduction

Long-lasting insecticidal nets (LLINs) and Indoor Residual Sprays (IRS) are the most widely used and effective vector control interventions for the control of malaria and other mosquito-borne diseases ( Bhatt *et al.*, 2015). Until recently, pyrethroids were the only class of insecticides used in LLINs ( World Health Organisation, 2015) with chlorfenapyr ( N’Guessan *et al.*, 2016) and pyriproxifen ( Ngufor *et al.*, 2014) being included more recently. Four additional classes of insecticides are currently used for IRS (organophosphates, carbamates, organochlorines and, recently, neonicotinoids; World Health Organisation, 2016). Increasing spread and intensity of insecticide resistance amongst African malaria vectors ( Ranson & Lissenden, 2016) threatens to reverse the progress that has been made in recent years ( World Health Organisation, 2019). To restore the efficacy of these vector control tools, insecticides that overcome existing resistance phenotypes and that are compatible for use in LLINs and IRS must be identified ( Hemingway *et al.*, 2016).

High-throughput screening of existing compound libraries and chemical synthesis programmes provide an opportunity to identify novel insecticides, but are costly, risky and take a long time from initiation to delivery as a vector control tool to the market ( Turner *et al.*, 2016). The cost of bringing a new crop protection product to market was estimated to be $286m in 2014 ( McDougall, 2016), and though the cost of developing a dedicated vector control insecticide would be lower, the total market for all public health insecticides may be only around $400m ( BMGF and BCG, 2007).

Repurposing of existing insecticides originally developed for non-vector control uses provides an alternative strategy for the development of vector control products containing active ingredients with new modes of action, benefiting from pre-existing registrations and manufacturing knowhow and, consequently, shorter development time, reduced risk and lower costs ( Hoppé *et al.*, 2016). Repurposing of active ingredients with modes of action that circumvent the resistance mechanisms associated with traditional neurotoxic insecticides such as pyrethroids, carbamates and organophosphates have been demonstrated as a means of delivering new insecticidal products, including Interceptor G2, a LLIN based on chlorfenapyr, developed by BASF for agriculture and urban pest control.

The Innovative Vector Control Consortium (IVCC) is a product development partnership established in 2005 with the aim of overcoming the market failures which inhibit the development of new insecticides for public health use by supporting the development of a range of new compounds and technologies for the control of mosquito populations in malaria endemic countries ( Hemingway *et al.*, 2006). In addition to IVCC’s efforts to develop completely novel compounds for vector control, this study was initiated to develop a laboratory bioassay cascade to screen existing agrochemical insecticides with no previous history of use in vector control that may have potential for LLIN and/or IRS uses and which offer new modes of action.

As the starting point for the testing cascade was insecticides that have already completed commercial development, a high throughput bioassay system, such as the larval screen developed by Matthews *et al.* (2018) was not necessary. Therefore, screening began with topical application to adult female mosquitoes. This served as a rapid first step to eliminate compounds with low intrinsic insecticidal activity against mosquitoes. However, as the potential uses being explored were for LLIN and IRS, all of the subsequent steps were based on insecticidal efficacy via tarsal contact. Tarsal contact assays were then run both with and without Mero, rapeseed oil, methyl ester(s) with an added emulsifier, which is a wetting agent developed for use with herbicides, hereafter referred to as RME. The addition of an adjuvant prevents the crystallisation of an insecticide on a glass surface was added to the evaluation of public health insecticides tested for IVCC since it seems to improve bioavailability and hence uptake by exposed insects ( Ohashi *et al.*, 2018). The inclusion of an additional step with an adjuvant was expected to increase the sensitivity of this testing cascade. Knock down of mosquitoes immediately after topical and tarsal exposure was scored in addition to mortality; although pyrethroids are known for their rapid effects on mosquito vectors, some compounds with different modes of action may be slower acting.

## Methods

### Compiling the list of compounds to include in the screening cascade

To evaluate the testing cascade, it was first necessary to compile a list of potential pesticides for inclusion in the project. The initial list included representative insecticides and acaricides from all Insecticide Resistance Action Committee (IRAC) mode of action (MoA) classes including those with unclassified mode of action ( IRAC, 2018). The initial list of 620 pesticidal active ingredients was reviewed to produce a shortlist of candidate compounds for testing. The criteria for determining which insecticides to include or eliminate from the list included a) elimination of insecticides from classes with current or historical vector control use (e.g. organochlorines, organophosphates, carbamates and pyrethroids); b) Elimination of active ingredients that did not have a current registration with at least one stringent regulatory authority (e.g. US EPA, EU Biocides, Japan etc.); and finally c) exclusion of arsenic based insecticides and cyclodienes on the grounds of safety, fumigants on the grounds of physiochemical properties, and insect growth regulators (juvenile hormone analogues, ecdysone antagonists etc.) because the focus of the project was on identifying compounds causing direct mortality.

**Figure 1.**
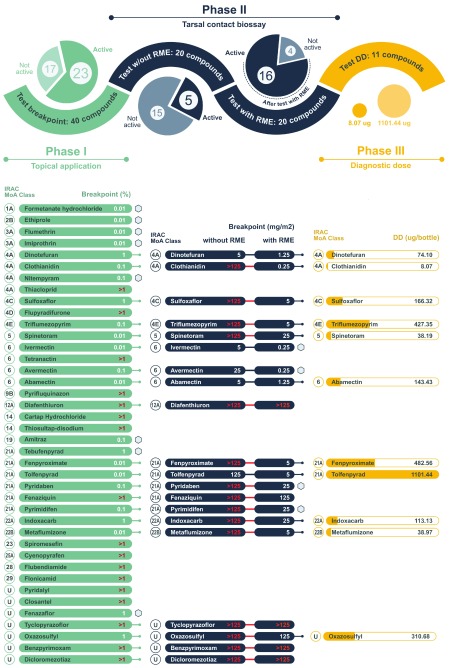
Testing cascade to screen existing insecticides for use against mosquito disease vectors, including key findings. Intrinsic insecticidal activity is measured by topical application of compound directly onto adult female *Anopheles gambiae* mosquitoes, activity through tarsal contact is measured in a bioassay with and without RME as an adjuvant, and relative potency is judged by determining the discriminating dose in CDC bottle bioassays. Activity in Phase I was defined as ≥80% mortality 24 hours after topical application, and the breakpoint denotes the concentration at which activity was first observed; ‘>1’ means that activity was not observed even when the highest concentration, 1% active ingredient, was applied. Activity in Phase II was defined as ≥80% mortality 24 hours after tarsal exposure, and the breakpoint denotes the concentration ay which activity was first observed; ‘>125’ means that activity was not observed even at the highest concentration tested, 125 mg/m ^2^. Compounds which were only active in tarsal contact bioassays (Phase II) in the presence and not the absence of RME are highlighted in red. Of 40 compounds from 20 IRAC MoA classes, and 7 compounds not listed or not classified by IRAC, 11 compounds are shortlisted for further investigation. The hexagon symbol denotes compounds which showed insecticidal activity during screening but which were not progressed for other reasons.

Where classes were represented by a number of active ingredients, one or two representative candidates were selected for initial screening. This resulted in a refined list of 40 compounds ( Figure 1).

Samples of technical-grade active ingredients were obtained either from commercial suppliers or via custom synthesis.

### Rearing mosquitoes for bioassays

All tests were carried out using 2-5-day-old non-blood-fed female mosquitoes of the insecticide-susceptible Kisumu strain of *Anopheles gambiae* s.s. reared in the Liverpool Insect Testing Establishment (LITE) insectaries at the Liverpool School of Tropical Medicine (LSTM) ( Edi *et al.*, 2012). Insectaries were maintained at 26°C ± 2°C and 80% relative humidity ± 10%. Larvae were fed ground fish food (Tetramin tropical flakes, Tetra, Blacksburg, VA, USA) and adults maintained on 10% sucrose solution fed *ad libitum*. For egg production, female adults were fed on human blood procured from the non-clinical blood product stock from the UK blood bank up until November 2016. After this point adult mosquitoes were fed on defibrinated horse blood supplied by TCS Biosciences. Mosquitoes were reared under a L12:D12 hour light:dark cycle with a 1 hour dawn and dusk.

### Intrinsic activity measured by topical application

A total of three replicates of ten mosquitoes were treated by topical application with solvent-only (negative control) and with 0.1% Permethrin in acetone (positive control) and with 0.01, 0.1 and 1% solutions of each compound solubilised in acetone, to identify compounds with intrinsic efficacy and to judge potency relative to Permethrin. Mosquitoes were anesthetized for 30 seconds using carbon dioxide, distributed across a Petri dish lined with filter paper (Whatman, Grade 1) with the dorsal thorax exposed, and placed onto a 4°C chill table (BioQuip products, Rancho Dominguez, CA). A 0.25-µl droplet of insecticide solution was applied to the dorsal thorax using a 1 cm ^3^ syringe and a hand-operated micro applicator (Burkhard Scientific, Uxbridge, UK). Mosquitoes were then transferred to holding cups, supplied with 10% sucrose solution, and held in a stability cabinet kept at 26°C ±2°C and 70% RH ±10% under a L12:D12 hour light: dark cycle. Mosquitoes were scored for knock down or mortality 24 hours and 48 hours after topical application, according to the World Health Organisation (WHO) definition ( World Health Organisation, 2018), a mosquito being classified as “dead” or “knocked down” if it is immobile or unable to stand or take off. After testing the first batch of compounds, an additional observation time was added to measure immediate effect on mosquitoes, with knock down being scored 60 minutes after topical application.

### Further screen for efficacy: tarsal contact assay with and without the addition of RME

A total of three replicates of ten mosquitoes were exposed to 0.05, 0.25, 1.25, 5, 25 and 125 mg/m ^2^ of each insecticide applied in acetone to glass Petri dishes (radius 2.5cm, area 19.635cm ^2^, SLS, Nottingham, UK), to identify compounds with efficacy via tarsal contact and to judge potency relative to Permethrin. Petri dishes treated with 0.05 and 5 mg/m ^2^ permethrin were also included as positive controls and Petri dishes treated with acetone-only as a negative control. A 500-µl aliquot of 0.000002, 0.00001, 0.00002, 0.0001, 0.0005, 0.002, 0.01 and 0.05% solutions of each insecticide solubilised in acetone were applied to glass Petri dishes and dishes placed onto an orbital shaker for 4 hours to provide an even coating of insecticide across the dish during evaporation of the acetone.

Bioassays were conducted immediately after the 4-hour drying period, at 26°C ±2°C and ambient humidity. A 25-ml plastic deli pot (Cater4you, High Wycombe, UK) with a hole melted through the base was fixed onto each Petri dish with Parafilm. Mosquitoes were introduced through the hole and, after exposing them to the treated surface for 30 minutes, they were aspirated out and transferred to holding cups. Mosquitoes were scored for knock down immediately after being placed into the cups, 30 minutes after the start of exposure. Mosquitoes were then held as for the topical application assay and scored for mortality 24 and 48 hours after exposure.

The assay was repeated for each compound with the addition of RME (Mero, Bayer, Reading, UK: 81.4% w/w rapeseed fatty acid esters and emulsifier ethoxy (7) tridecanol) in combination with each insecticide. A 0.392 mg/ml RME in acetone solution (a concentration based on a typical field application rate when used with herbicides) was used to prepare insecticide dilutions, which were applied to the glass Petri dishes to give a final application rate of 100 mg/m ^2^ RME and the bioassay conducted as described above.

### Judging relative potency by determining discriminating doses

The discriminating dose (DD) of 11 compounds (dinotefuran, clothianidin, sulfoxaflor, triflumezopyrim, spinetoram, abamectin, fenpyroximate, Tolfenpyrad, indoxacarb, metaflumizone and oxazosulfyl) was established using a variation of the CDC bottle bioassay test ( Brogdon & Chan, 2010). Wheaton bottles (250 ml, Fisher Scientific, Loughborough, UK) were coated with active ingredient dissolved in acetone as described, but with the addition of with 1.5 mg/bottle of RME, or 53.6 mg/m ^2^. This lower concentration was validated in a preliminary assay (data not shown) after the rate of 100 mg/m ^2^ used for the tarsal contact assay produced stickiness and higher than acceptable negative control mortality in the bottle bioassay. Bioassays were conducted a minimum of an hour and no more than 24 hours after coating, at 26°C ±2°C and 70% ± 10% relative humidity. A total of 25 female mosquitoes were added to the test bottles alongside a negative control bottle containing acetone and RME only, and a positive control bottle containing 20 µg/bottle permethrin applied in acetone but without RME. After a 60-minute exposure, mosquitoes were transferred from the bottles into holding cups, and scored as knocked down or dead if they are immobile or unable to stand as described by Brogdon & Chan (2010). Mosquitoes were held as for topical and tarsal contact assays, and mortality scored 24 hours after exposure. Mosquitoes were exposed to different concentrations (reported in µg/bottle) of compounds until data was collected from concentrations which produced mortality values covering the whole range from 0–100%. These data were used to calculate the DD of each compound.

### Data analysis

Percentage knock down and percentage mortality were calculated from the total number of mosquitoes in each replicate of the topical and tarsal assays counted at the end of the experiments. If mortality in the topical application or tarsal contact test negative control was between 5 and 20% mortality in test replicates was corrected for negative control mortality using Abbott’s formula ( Abbott, 1925), with negative values reported as 0% mortality. If control mortality was above 20% at 24 hours after exposure the test was repeated. Breakpoints were determined as being the lowest concentration at which 80% or more of mosquitoes were killed within 24 hours of exposure. Compounds which failed to reach 80% mortality at 24 hours after topical application or tarsal contact exposure with RME were rejected from further testing in the cascade.

To calculate a DD for each compound, 24-hour mortality data was included from at least *n*=50 mosquitoes exposed to each concentration used in the analysis. Data from concentrations below the highest tested concentration that gave 0% mortality 24 hours after exposure, above the lowest tested concentration that gave 100% mortality, and those with an *n* value lower than 50 were excluded from data analysis. The number of subjects *n* and the number of responders *r* were aggregated across the replicates for each concentration, and these data were analysed to determine LC _95_ values for each compound using PoloPlus software (Version 2.1, LeOra Software), via log-probit analysis ( Robertson *et al.*, 1980). This calculated LC _95_ value was then multiplied by three to give a discriminating dose (LC _95_×3=DD), and compounds were ranked for relative potency: a low DD indicates a higher potency. This differs from the standard WHO discriminating dosages which are defined as being twice the experimentally derived 100% lethal concentration (LC _100_ value) of a reference susceptible strain ( World Health Organisation, 1998).

## Results

**Figure 2.**
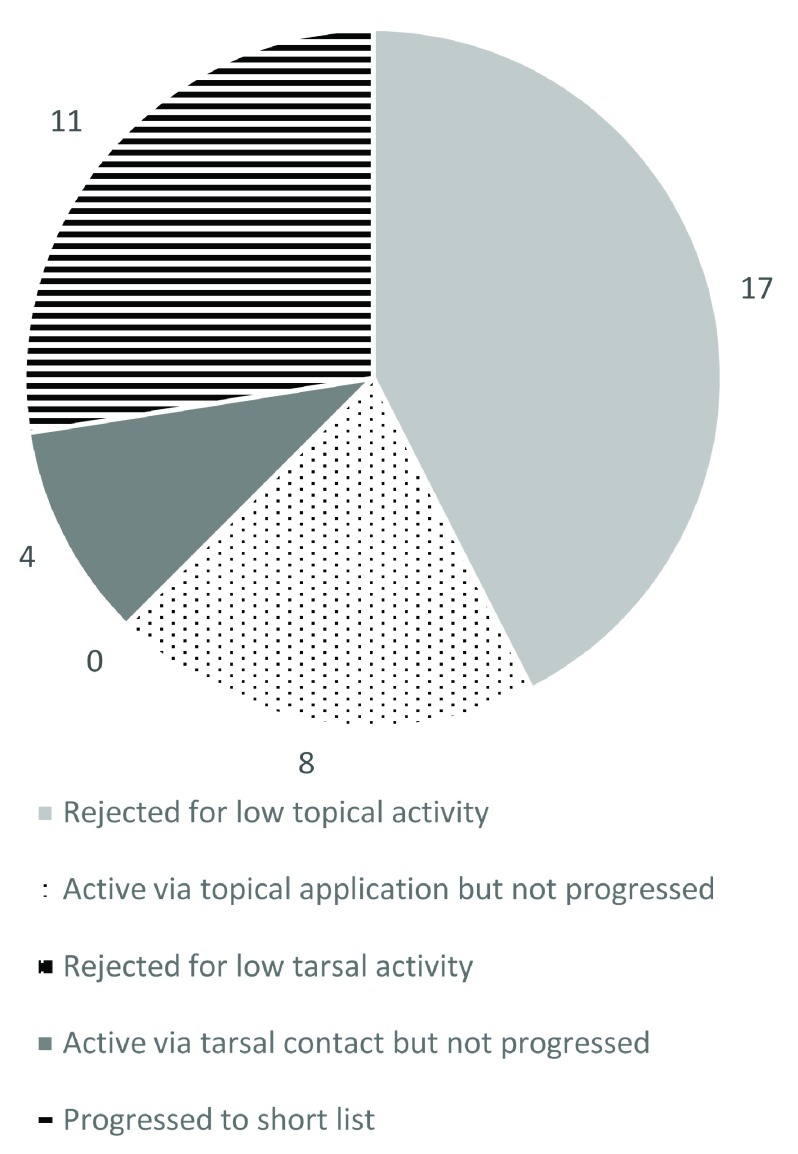
Fate of the 40 compounds taken through the testing cascade. In general, compounds which were active (≥80% mortality 24 hours after exposure) in topical application were progressed to tarsal contact assays, and those active at this step were progressed to measure relative potency through determining their discriminating dose. No compounds were therefore rejected for low tarsal activity. However, of the 23 compounds which were active when applied topically, not all were progressed to tarsal contact testing due to a range of considerations including physicochemical properties and regulatory challenges foreseen in their development. Of the compounds which were active in tarsal contact assays, only a representative compound from each IRAC MoA class were progressed, with the exception of fenpyroximate and tolfenpyrad, both from class 21A.

Forty compounds were included in the testing cascade, first tested for activity when applied topically, then for activity on tarsal exposure alone and in the presence of RME as an adjuvant, to identify a shortlist of 11 compounds which have potential for use to control *Anopheles* vectors of disease, which were then ranked for relative potency by calculating their DD. An overview of the data is presented in Figure 1. During the completion of the study additional information was gathered about the compounds of interest, beyond entomological activity, and some compounds were not progressed in the cascade despite demonstrating efficacy, as described below and summarised in Figure 2.

### Intrinsic activity measured by topical application

Mortality observed 24 hours after topical application of 40 compounds is presented in Table 1, together with knock down observed 60 minutes after topical application where these data were collected. Major differences were evident between the insecticidal efficacy of test chemicals in terms of the level of knock down and mortality they caused, demonstrating the sensitivity of the method. Activity was not determined relative to the positive control as only a single concentration of permethrin was applied (0.1%). The positive control consistently resulted in 100% mortality.

**Table 1.** Effect of topical application of forty compounds on adult female *Anopheles gambiae*. Knock down and mortality was observed 60 minutes and 24 hours after topical application, respectively, and were corrected for negative control mortality using Abbott’s formula ( Abbott, 1925). UN denotes a compound listed by IRAC (2018) with unknown or uncertain mode of action. Compounds indicated as being unclassified have not been listed by IRAC (2018). ND indicates a data point which was not collected.

IRAC Class	Compound	Mean KD 60 minutes (%)			Mean Mortality 24 hours (%)			Breakpoint (% AI)
		0.01%	0.1%	1%	0.01%	0.1%	1%	
**1A**	Formetanate hydrochloride	ND	ND	ND	81.50	86.20	90.00	0.01
**2B**	Ethiprole	ND	ND	ND	100.00	100.00	100.00	0.01
**3A**	Flumethrin	6.67	86.67	96.88	96.70	100.00	96.70	0.01
	Imiprothrin	ND	ND	ND	96.70	96.70	100.00	0.01
**4A**	Dinotefuran	74.42	100	100	65.13	0.00	100.00	1
	Clothianidin	80.65	81.25	93.1	100.00	96.49	96.49	0.1
	Nitenpyram	96.88	100	100	96.56	100.00	100.00	0.1
	Thiacloprid	63.33	81.25	86.67	19.00	0.00	42.33	>1
**4C**	Sulfoxaflor	37.68	50	93.33	58.52	78.61	100.00	1
**4D**	Flupyradifurone	ND	ND	ND	48.20	33.30	73.60	>1
**4E**	Triflumezopyrim	29.17	64.44	92.96	55.50	89.12	100.00	0.1
**5**	Spinetoram	90	76.67	90	92.56	100.00	100.00	0.01
**6**	Ivermectin	13.1	35.69	60.98	100.00	100.00	100.00	0.01
	Tetranactin	ND	ND	ND	0.00	0.00	3.54	>1
	Avermectin	ND	ND	ND	73.00	100.00	100.00	0.1
	Abamectin	0	19.35	35.71	100.00	100.00	95.80	0.01
**9B**	Pyrifluquinazon	86.67	45.16	80.77	46.37	18.11	77.85	>1
**12A**	Diafenthiuron	3.23	85.19	72.73	19.31	11.68	20.50	>1
**14**	Cartap Hydrochloride	77.42	100	100	13.40	30.00	40.00	>1
	Thiosultap-disodium	9.09	6.67	44.44	19.22	0.00	42.33	>1
**19**	Amitraz	ND	ND	ND	97.50	86.70	79.30	0.1
**21A**	Tebufenpyrad	41.38	60	74.07	0.58	34.60	82.93	1
	Fenpyroximate	96.77	100	100	85.22	96.22	82.36	0.01
	Tolfenpyrad	47.58	90.91	100	100.00	100.00	100.00	0.01
	Pyridaben	ND	ND	ND	42.00	94.00	76.00	0.1
	Fenaziquin	3.33	5.56	31.85	3.98	31.33	70.42	>1
	Pyrimidifen	45.45	96.67	100	64.63	100.00	100.00	0.1
**22A**	Indoxacarb	3.7	3.33	0	68.15	76.67	100.00	1
**22B**	Metaflumizone	14.29	27.59	25	92.90	96.60	96.40	0.01
**23**	Spiromesefin	ND	ND	ND	0.00	0.00	10.00	>1
**25A**	Cyenopyrafen	0	0	0	10.00	3.33	6.66	>1
**28**	Flubendiamide	3.33	3.33	6.67	16.70	16.70	3.30	>1
**29**	Flonicamid	3.33	10.34	56.67	6.70	13.80	30.00	>1
**UN**	Pyridalyl	0	0.33	0	0.00	0.00	0.78	>1
**Unclassified**	Closantel	6.67	10	10	0.00	7.11	32.08	>1
	Fenazaflor	3.33	82.76	71.88	0.00	77.81	89.92	1
	Tyclopyrazoflor	14.44	35.02	38.18	38.89	46.46	66.67	>1
	Oxazosulfyl	25.45	25.45	50.34	70.91	74.85	96.67	1
	Benzpyrimoxam	0	0	3.33	0.65	0.00	8.53	>1
	Dicloromezotiaz	0	0	0	0.00	0.00	2.54	>1

A total of 40 compounds were screened in this initial step in the testing cascade, of which 17 failed to reach the 80% mortality threshold 24 hours after application of the highest dose. Of these, 13 were eliminated at this stage but five compounds (diafenthiuron, fenazaquin, tyclopyrazoflor, benzpyrimoxan and dicloromezotiaz) were taken forward to testing in the tarsal contact assays in order to validate topical application as a first step in the testing cascade. There were ten compounds that reached the threshold of activity at 0.01%, the lowest concentration tested; six reached the threshold at the intermediate dose of 0.1%. On reviewing these results and other available information about the compounds, 15 of these 23 compounds were taken forward to the next step of the testing cascade. 

There were eight compounds which showed activity in the topical application screen that were not taken forward for further testing for reasons other than insecticidal efficacy. Tebufenpyrad was rejected because four other active mitochondrial complex I electron transport inhibitors were already being taken forward. Fenazaflor was rejected as it was no longer commercially available. Ethiprole and nitenpyram showed acceptable activity with topical application but were not taken forward in the testing cascade due to their high water solubility as this made them unsuitable for use in LLINs, the priority development area for IVCC at the current time, but may have potential for use in IRS. Flumethrin and imiprothrin were included in this initial topical application screen as potential alternative pyrethroids. They were active at the lowest concentration tested and may be useful in providing alternatives to existing pyrethroids used on LLINs. However, they did not meet the key criteria of this study, being alternatives to pyrethroids with novel modes of action for vector control, and so were not taken forward at this time. Formetanate hydrochloride and amitraz were rejected despite being active in topical application due to perceived regulatory challenges.

Mortality was assessed at 24 and 48 hours after topical application of the test compounds. However, only seven compounds gave more than a 20% increase in mortality between these two time points, and in each case only with one of the three concentrations tested ( Lees *et al.*, 2019). For four compounds the breakpoint (concentration giving 80% mortality) decreased between 24-hour and 48-hour observations: dinotefuran from 1 to 0.01%; sulfoxaflor from 1 to 0.1%; and pyrifluquinazon and tyclopyrazoflor which did not reach 80% mortality based on 24-hour observation, but by 48 hours post-exposure the threshold was reached at the highest concentration, 1 %. 

For 31 compounds, knockdown was assessed 60 minutes after topical application in addition to assessment of mortality, and breakpoints were determined. Four compounds (spinetoram, fenpyroximate and pyrimidifen) had the same breakpoint for knock down and mortality, indicating relatively rapid action, whilst for nine compounds, a lower breakpoint was observed at 24-hour mortality, indicating slow action. Within this latter list of compounds, ivermectin, abamectin and metaflumizone stand out as being effective in terms of mortality but gave very low knock down rates. The breakpoint was lower when scored at 60 minutes in nine compounds, indicating some recovery from knock down during the subsequent holding period: thiacloprid, pyrifluquinazon, diafenthiuron and cartap hydrochloride were particularly active in terms of knock down but failed to achieve 80% mortality at 24 hours.

### Further screen for Efficacy: Tarsal contact assay with and without the addition of RME

A total of 16 compounds from nine IRAC MoA classes, plus four which are unclassified by IRAC (20 compounds in total), were taken forward to be tested in tarsal contact bioassays with and without RME as an adjuvant. Knockdown 60 minutes after exposure and mortality 24 hours after exposure, in the presence and absence of RME, is presented in Table 2. Only five compounds, dinotefuran, avermectin, abamectin, tolfenpyrad and ivermectin achieved 80% mortality 24 hours post-exposure in the absence of RME, at any of the doses tested. 

**Table 2.** Effect of tarsal contact with 20 compounds, with/without RME, on adult female *Anopheles gambiae*. There were 20 compounds applied in acetone to a glass plate and allowed to dry before mosquitoes were exposed to the surface and scored for knock down and mortality 60 minutes and 24 hours, respectively, after exposure. Percentages have been corrected for negative control mortality using Abbott’s formula ( Abbott, 1925). Compounds indicated as being unclassified have not been listed by IRAC (2018). ND indicates a data point which was not collected.

Compound (IRAC Class)	Concentration (mg AI/m ^2^)	Compound alone		Compound + RME		Breakpoint with RME (mg AI/m ^2^)
		Mean % Knock Down	Mean % Mortality	Mean % Knock Down	Mean % Mortality	
**Dinotefuran** **(4A)**	125	100.00	100.00	100.00	100.00	1.25
	25	79.17	100.00	93.75	100.00	
	5	31.03	86.21	97.14	100.00	
	1.25	28.00	64.00	93.33	100.00	
	0.25	6.06	15.15	14.29	78.57	
	0.05	10.00	0.00	3.85	42.31	
**Clothianidin** **(4A)**	125	11.99	35.71	68.00	100.00	0.25
	25	12.95	31.25	89.45	100.00	
	5	59.03	67.65	96.38	100.00	
	1.25	60.71	60.00	82.94	100.00	
	0.25	3.94	6.90	40.00	96.55	
	0.05	9.60	9.38	0.00	78.13	
**Sulfoxaflor** **(4C)**	125	6.36	70.91	82.14	96.43	5
	25	0.00	40.94	96.55	100.00	
	5	0.00	26.36	35.71	85.71	
	1.25	0.00	6.67	0.00	58.62	
	0.25	0.00	3.03	6.90	10.34	
	0.05	5.90	0.00	0.00	11.11	
**Triflumezopyrim** **(4E)**	125	ND	ND	ND	ND	5
	25	36.67	50.00	60.71	96.97	
	5	6.36	20.00	9.09	81.82	
	1.25	10.37	35.56	0.00	62.22	
	0.25	0.00	0.00	8.82	25.07	
	0.05	0.00	0.00	6.67	6.73	
**Spinetoram** **(5)**	125	7.69	73.08	0.00	100.00	25
	25	9.68	29.03	22.50	100.00	
	5	3.33	10.00	0.00	71.82	
	1.25	7.69	7.69	6.58	45.10	
	0.25	7.41	3.70	7.14	40.00	
	0.05	0.00	6.45	0.00	0.36	
**Ivermectin** **(6)**	125	5.00	100.00	0.00	100.00	0.25
	25	5.00	100.00	0.00	100.00	
	5	0.00	100.00	10.00	100.00	
	1.25	0.00	70.00	0.00	95.00	
	0.25	0.00	10.00	0.00	95.00	
	0.05	0.00	5.00	5.00	60.00	
**Avermectin** **(6)**	125	20.94	100.00	ND	ND	0.25
	25	2.00	100.00	37.50	100.00	
	5	9.00	37.00	21.21	100.00	
	1.25	3.45	3.45	14.71	97.06	
	0.25	0.00	0.00	39.29	92.86	
	0.05	0.00	0.00	0.00	24.14	
**Abamectin** **(6)**	125	17.86	96.43	0.00	100.00	1.25
	25	13.33	100.00	46.88	100.00	
	5	6.90	89.66	35.48	100.00	
	1.25	10.34	41.38	55.88	94.12	
	0.25	3.33	20.00	9.09	66.67	
	0.05	0.00	0.00	13.79	13.79	
**Diafenthiuron** **(12A)**	125	0.00	3.23	ND	ND	>125
	25	6.90	10.34	0.00	48.17	
	5	0.00	6.45	0.00	0.00	
	1.25	10.00	10.00	1.59	1.02	
	0.25	3.45	0.00	0.00	0.00	
	0.05	0.00	0.00	0.00	0.00	
**Fenpyroximate** **(21A)**	125	22.73	59.09	100.00	100.00	5
	25	12.90	16.13	100.00	96.28	
	5	9.68	6.45	85.03	82.01	
	1.25	7.14	21.43	45.63	61.66	
	0.25	8.00	4.00	13.94	10.05	
	0.05	15.38	3.85	2.71	31.64	
**Tolfenpyrad** **(21A)**	125	70.37	100.00	86.21	100.00	5
	25	12.50	3.13	91.18	97.06	
	5	7.41	11.11	82.86	88.57	
	1.25	7.14	7.14	53.33	46.67	
	0.25	0.00	3.33	43.75	18.75	
	0.05	3.23	0.00	24.14	17.24	
**Pyridaben** **(21A)**	125	42.72	14.49	75.06	90.00	25
	25	43.31	7.05	96.44	100.00	
	5	23.78	0.00	69.94	68.75	
	1.25	32.37	0.00	38.45	36.36	
	0.25	17.86	0.00	0.00	3.23	
	0.05	34.17	38.83	9.81	15.63	
**Fenaziquin** **(21A)**	125	20.88	21.55	80.00	80.00	125
	25	20.83	0.00	63.64	33.33	
	5	8.33	3.33	17.65	5.88	
	1.25	9.44	6.11	3.23	3.23	
	0.25	0.00	6.67	3.57	7.14	
	0.05	3.03	0.00	3.23	0.00	
**Pyrimidifen** **(21A)**	125	54.07	46.67	90.63	81.25	25
	25	50.00	6.67	100.00	100.00	
	5	6.67	6.67	51.61	54.84	
	1.25	0.00	0.00	17.86	3.57	
	0.25	0.00	0.00	10.00	10.00	
	0.05	0.00	0.00	17.86	14.29	
**Indoxacarb** **(22A)**	125	3.03	1.55	3.33	100.00	25
	25	3.33	10.00	3.23	93.55	
	5	0.00	0.00	0.00	76.92	
	1.25	2.78	2.80	3.45	31.03	
	0.25	0.00	0.00	6.45	6.45	
	0.05	0.00	0.00	0.00	0.00	
**Metaflumizone** **(22B)**	125	3.33	3.33	7.41	64.55	5
	25	0.00	3.33	3.33	100.00	
	5	0.00	0.00	6.67	100.00	
	1.25	0.00	3.33	0.00	61.11	
	0.25	0.00	3.33	3.33	16.67	
	0.05	0.00	6.67	3.33	3.33	
**Tyclopyrazoflor** **(Unclassified)**	125	33.33	73.33	64.29	64.29	>125
	25	23.70	68.89	28.13	59.38	
	5	10.00	40.00	22.58	35.48	
	1.25	3.03	3.03	17.24	13.79	
	0.25	6.67	7.04	0.00	0.00	
	0.05	0.00	0.00	0.00	6.45	
**Oxazosulfyl** **(Unclassified)**	125	3.33	3.33	36.67	100.00	5
	25	3.33	6.36	43.33	100.00	
	5	0.00	13.89	50.00	90.00	
	1.25	0.00	0.00	56.67	60.00	
	0.25	3.33	0.00	3.33	3.33	
	0.05	0.00	0.00	0.00	6.90	
**Benzpyrimoxam** **(Unclassified)**	125	3.33	6.67	3.13	0.00	>125
	25	0.00	3.33	0.00	7.14	
	5	0.00	0.00	0.00	0.00	
	1.25	0.00	0.00	0.00	7.14	
	0.25	0.00	3.33	0.00	0.00	
	0.05	0.00	6.67	0.00	3.57	
**Dicloromezotiaz** **(Unclassified)**	125	0.00	3.33	0.00	13.33	>125
	25	0.00	0.00	0.00	3.33	
	5	0.00	0.00	6.67	6.67	
	1.25	0.00	0.00	0.00	6.06	
	0.25	0.00	0.00	19.35	25.81	
	0.05	0.00	0.00	24.14	24.14	

An additional 11 compounds, from eight different IRAC MoA classes plus one unclassified compound, reached 80% mortality in the tarsal test in the presence of RME. All compounds which achieved 80% mortality in tarsal bioassays did so at a lower concentration in the presence of the adjuvant RME. 

Of the 16 compounds which were thus judged to be active in tarsal contact assays, 11 were taken forward to the next stage in the testing cascade. Fenazaquin had previously been rejected due to low activity at topical application and since it was active only at the highest concentration in tarsal assay with RME it was not progressed. The other four were not progressed since they were represented by other compounds in the same IRAC class. Tolfenpyrad and fenpyroximate were taken forward as the most efficacious mitochondrial complex I electron transport inhibitors, and abamectin was taken forward to represent the avermectin class due to the availability of commercial formulations. 

The five compounds which did not show activity in the topical application screen but which were included for method validation purposes also did not reach the 80% mortality threshold in tarsal contact assays either with or without the addition of RME, with the exception of fenazaquin which was active only at the highest concentration tested (125 µg/bottle) and only with the addition of RME.

Initially, mortality was assessed 24 and 48 hours after exposure. However, no compound gave more than a 10% increase in mortality between the 24-hour and 48-hour assessments in any tarsal assay with or without the addition of RME. Therefore, a 48-hour mortality assessment was not included for all compounds and mortality data is presented here at the 24-hour assessment only ( Table 2). 

Compounds were defined as fast acting if more than 80% knock down was observed immediately post-exposure at the breakpoint concentration. Based on tarsal contact in the absence of RME, only dinotefuran was judged to be fast acting, but with the addition of RME, fenpyroximate, pyridaben, fenazaquin, pyrimidifen and tolfenpyrad were also judged to be fast acting (although knock down was not scored for oxazosulfyl or Indoxacarb). Of these, fenpyroximate and pyrimidifen had also been judged to be fast acting through topical application.

### Judging relative potency by calculating DD

**Figure 3.**
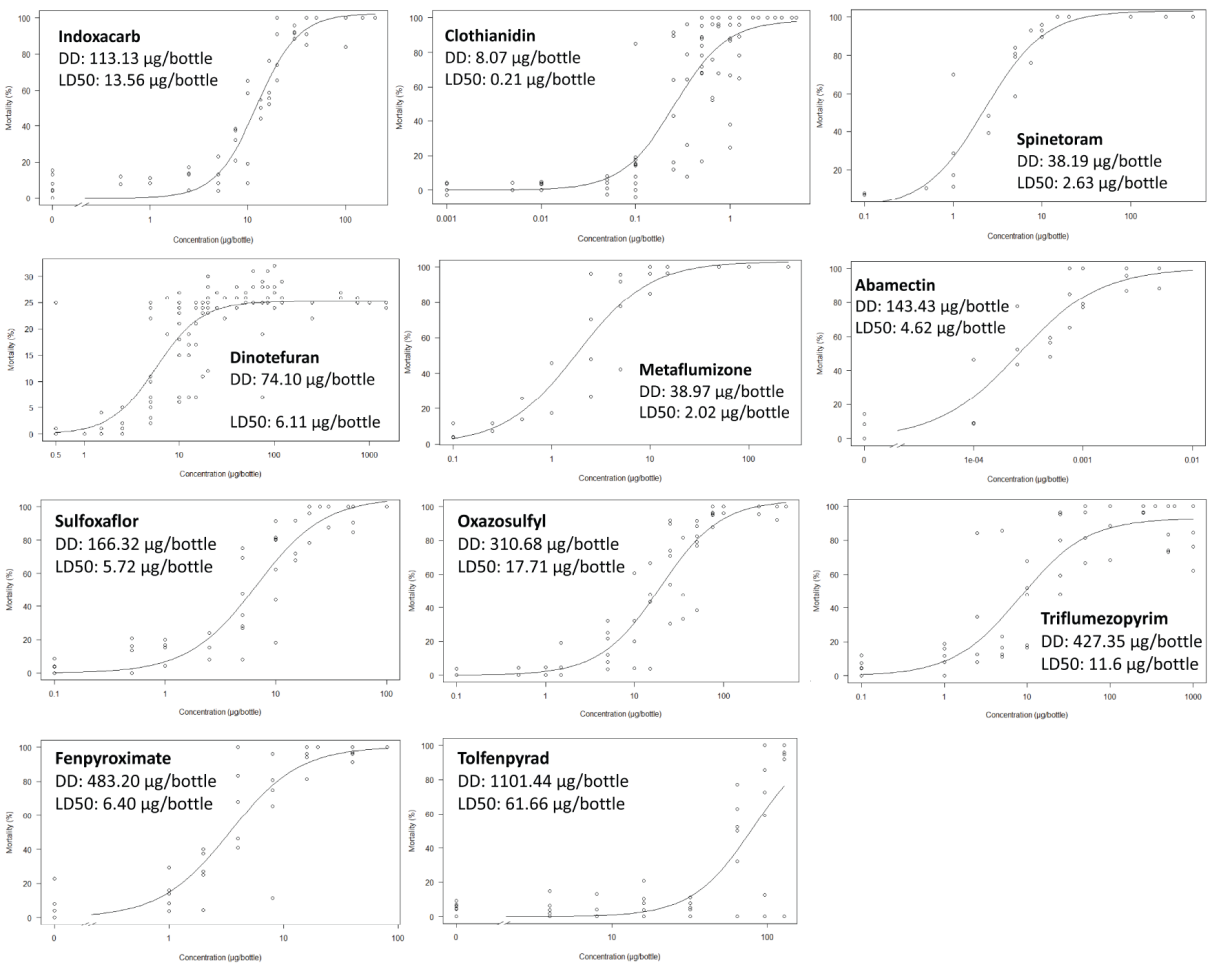
The 24-hour mortality dose response curves and discriminating doses of the 11 shortlisted compounds. Dose response curves were produced by measuring mortality of adult female *Anopheles gambiae* mosquitoes exposed to dried deposits of compounds applied in acetone to the inside of a bottle, in a version of the CDC bottle bioassay. Discriminating dose is calculated as the LC _95_ multiplied by 3.

**Figure 4.**
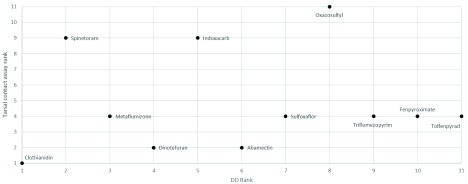
Relative potency of compounds assessed by discriminating dose and tarsal contact assay with RME. Compounds in the shortlist ranked from highest (1) to lowest (11) potency according to the breakpoint concentration in the tarsal contact assay with RME is plotted against the rank assigned according to the DD. Where compounds have the same breakpoint they were assigned the same rank and ranks skipped correspondingly.

**Figure 5.**
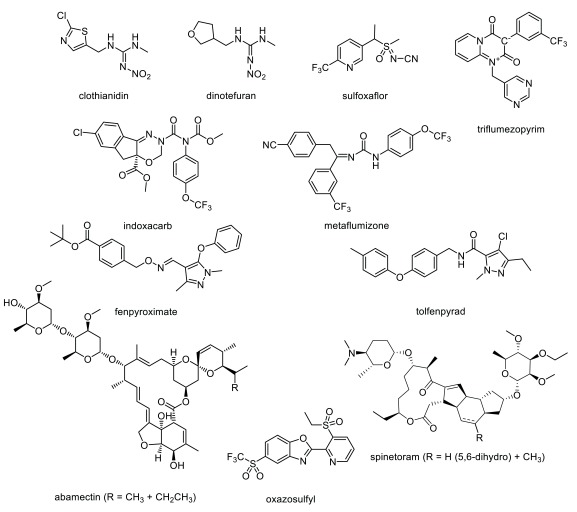
Chemical structures of the shortlisted compounds. From the 40 compounds taken through the testing cascade for repurposed pesticides, 11 are highlighted as potentially suitable for use against mosquito vectors of malaria in an insecticide treated net. Their chemical structures are presented here. Compounds are arranged by IRAC MoA class, class 4 Nicotinic acetylcholine receptor competitive modulators on the first row, class 22 Voltage-dependent sodium channel blockers on the second, and mitochondrial complex 1 electron transport inhibitors on the third. Abamectin is Class 6, a glutamate-gated chloride channel (GluCl) allosteric modulator, spinetoram is Class 5, a nicotinic acetylcholine receptor (nAChR) allosteric modulator – Site I. Oxazosulfyl is not listed by IRAC.

A total of 11 compounds were shortlisted after review of the results from tarsal contact assays. Discriminating doses, calculated from 24-hour mortality data from bottle bioassays in the presence of RME and ranked in order of potency, are shown in Figure 3. The DDs (three times the LC _95_ values from log-probit analysis) ranged from 8.07 µg/bottle for clothianidin to 1101.44 µg/bottle for tolfenpyrad. Clothianidin had a significantly greater potency than any other compound on the short list, followed by spinetoram and metaflumizone which were significantly more potent than all other compounds except dinotefuran (95% CI, log-probit analysis). In order to compare these results with results from the previous step in the testing cascade, compounds were ranked by DD and by breakpoint in the tarsal contact assay with RME, and these ranks plotted against each other ( Figure 4) to show the correlation. The chemical structures of these 11 compounds are shown in Figure 5.

## Discussion

### Evaluating the testing cascade

The testing cascade described here was designed to fulfil two purposes: firstly, to identify existing crop protection and animal health insecticides which have intrinsic activity against mosquito vectors of disease and potential for use in public health; and secondly to assess their efficacy via tarsal contact. The potential for improved activity in an appropriate formulation was explored through the inclusion of an adjuvant, RME. Finally, compounds were ranked by relative potency by determining their discriminating dose. A high throughput screen of a large panel of compounds, such as the 12 well plate assay of Hoppé *et al*. (2016), was replaced in this instance by an initial paper exercise to identify existing pesticides with potential for repurposing for mosquito control based on available information including toxicology and commercial production. Forty compounds were taken through the subsequent testing cascade to explore its usefulness to facilitate evidence-based decision making on which compounds should be investigated further. Following inclusion of factors other than insecticidal efficacy, including safety, regulatory environment and availability, a shortlist of 11 compounds with potential for formulation into mosquito vector control products is proposed.

Topical application of active ingredients in a volatile solvent does not reflect the way that mosquitoes would be exposed to insecticides in an LLIN or IRS product and, in this sense, is a less realistic screen than the tarsal contact assay. However, it is recommended by WHO as the first step in screening adulticides for possible inclusion in indoor residual sprays (IRS) or mosquito nets to identify compounds with intrinsic activity against mosquito vectors ( World Health Organisation, 2006). Topical application is suitable as a rapid first stage in a screening cascade, where screening large numbers of compounds through tarsal contact may not be logistically feasible. Much smaller quantities of compound are required for topical application, which is an important consideration when compounds can be costly to purchase or to synthesise.

The WHO guidelines recommend tarsal exposure of mosquitoes to compound applied to filter paper in a WHO tube assay as the next step in screening potential new insecticides ( World Health Organisation, 2006). However, to minimise the quantities of compound required, a method that involved exposing mosquitoes to small treated Petri dishes was developed to determine efficacy through tarsal contact, the typical route of exposure of mosquitoes to the chemistries used in IRS and LLINs. 

Of the five compounds which failed to reach the activity threshold in the topical application screening, but which were taken forward to tarsal testing to validate the screening cascade as a whole, all showed little or no activity in the tarsal contact assay except Fenazaquin which was active only at the highest concentration tested. It does highlight a potential issue with a step-wise testing cascade: a compound may be active via one route of exposure but not be progressed due to low activity at an earlier testing stage. Of the 14 compounds which reached the 80% threshold for activity in topical application, all were also active in the tarsal contact assays in the presence of an adjuvant, although not always without adjuvant. A good correlation was seen between the results of the two testing stages, with activity of active ingredients when applied topically being a good predictor of tarsal contact activity, with compounds having very low intrinsic activity being screened out in the process. The final stage in this testing cascade, the calculation of DD based on 24-hour mortality in mosquitoes exposed to a treated bottle, was included to provide robust data on the relative potency of the shortlisted compounds. Combined with information on cost of goods information, this provides information on the cost-efficacy of a compound. The WHO recommends the use of twice the calculated LC _99.9_ values determined by baseline susceptibility testing against a susceptible laboratory strain or a susceptible field population of mosquitoes as the DD ( World Health Organisation, 2018). However, using the LC _99.9_ is prone to error since the confidence intervals are typically wider at the extreme lower and upper range of LC values, and a much greater number of replicates are required to achieve an accurate estimate ( Robertson *et al.*, 1980). For this reason, a DD of three times the calculated LC _95_ was used in this screen.

The inclusion of a concentration range in the tarsal contact assay was designed to give more quantitative data and allow compounds to be selected for progression based on level of activity, with a breakpoint concentration for each being used as a measure of potency. However, there was a poor correlation between this breakpoint and the DD for the final list of proposed compounds, and activity in the tarsal contact assay was a poor predictor of DD. Clothianidin had the lowest DD (i.e. was the most potent) of the compounds on the shortlist and reached 80% mortality 24 hours after exposure to the lowest dose used in the tarsal contact assay with RME, which none of the other shortlisted compounds achieved. However, this does not hold true with the other compounds. For example, spinetoram was the second most potent of the compounds in terms of DD but was relatively inactive in the tarsal contact assay with RME. There are some key differences in the format of these assays which may go some way to explaining this discrepancy. For example, for a compound having a higher volatility, the greater quantity of compound applied to the inside of the sealed bottles used in the bottle bioassay may have a disproportionate effect relative to the smaller surface area and more ventilated environment of the tarsal contact assay. The small number of mosquitoes used in each tarsal contact assay may also lead to a certain level of ‘noise’ in the data not seen with the larger scale of the dose response assays which along with the more robust analysis, means that the ranking of compounds for potency is likely to be more accurate based on the DD analysis than on the earlier stages in the testing cascade.

When viewed together, the data collected on the compounds evaluated through this screening cascade suggest that a topical application screen is sufficient to eliminate compounds without sufficient intrinsic activity against the target mosquito species, and that calculating the DD is the most informative subsequent assay for the selection of the most potent compounds for further development. This more streamlined screening cascade, without the inclusion of the tarsal contact assay, may be an efficient way to identify compounds with potential for development for LLIN or IRS products. Other tests may be required to identify compounds suitable for use in other product classes. Flupyradifurone, for example, was rejected based on low efficacy in the topical screen but has since been developed into a space spray product by Bayer Vector Control, Fludora ^®^ Co-Max EW, in combination with Transfluthrin. It may be that atomisation of the compound into small droplets that contact across a greater area of the mosquito serves to increase potency, and that inclusion of a screening methodology such as a wind tunnel may be valuable for those compounds which are not shown to be active in topical application. Volatile insecticides with potential for use in commercial household insecticide emanators might be better tested in an assay which exposes mosquitoes without physical contact with a compound.

### The importance of including an adjuvant

Where tarsal contact assays are to be used to screen compounds for activity against mosquitoes, this study demonstrates how important it is for an adjuvant to be applied to the treated surface along with the active ingredient. Of the 11 compounds shortlisted by following this testing cascade, eight would have been rejected as having insufficient tarsal efficacy when applied to bottles with acetone alone but, with the addition of the adjuvant RME, reached the acceptable threshold of activity: sulfoxaflor, triflumezopyrim, spinetoram, fenpyroximate, oxazosulfyl, clothianidin and indoxacarb. Each of these compounds, with the exception of oxazosulfyl, which was not included in their screen, were rejected by Hoppé *et al* (2016) as having too low an activity against *Aedes aegypti*, and in some cases also against *Anopheles stephensi*. Although differential activity of insecticides against different species is sometimes seen, the inclusion of an adjuvant is a potential explanation, or at least partial explanation, for this lack of correlation between studies. Of particular note on this list is clothianidin, which is the active ingredient in Sumishield 50WG and Fludora Fusion, IRS products produced by Sumitomo Chemicals and Bayer Crop Science, respectively, which was found to be inactive against *Aedes aegypti* though not tested against *An. stephensi* ( Hoppé *et al.*, 2016). 

Susceptibility testing of clothianidin is performed by Sumitomo Chemical using the CDC bottle bioassay ( Brogdon & Chan, 2010) with the addition of 500 µg/bottle Span 80, a surfactant. The addition of Span 80 is shown to prevent the formation of large crystals of clothianidin on the glass surface which form particularly at high concentrations and are correlated with the reduction of mortality in exposed *An. gambiae* ( Ohashi *et al.*, 2018). Ohashi *et al*. (2018) calculated a DD for *Anopheles gambiae* of 20 µg/bottle for clothianidin in the presence of Span 80 with a 2-hour exposure time, and a much lower potency was measured in the absence of the surfactant. Since the current study calculated the DD to be less than half this with a 60-minute exposure it is possible that RME may be a more effective adjuvant than Span 80, although variations between experimental conditions cannot be discounted as an alternative explanation. In its current multi-centre study to identify discriminating doses, the WHO is including RME in the methodology for clothianidin, as well as for flupyradifurone and imidacloprid (Corbel, personal communication). 

The activity of a suspension concentrate formulation of indoxacarb has been demonstrated on treated nets against *Anopheles gambiae* and *Culex quinquefasciatus* ( Oxborough *et al.*, 2015a), which again would have been rejected following testing without the addition of RME but was included in the final shortlist of proposed compounds in this study. The neonicotinoid dinotefuran has been shown to be toxic against strains of *Anopheles gambiae* Giles, *Culex quinquefasciatus* Say, and *Aedes aegypti* L carrying commonly found resistance mechanisms, and may be even more effective against mosquitoes resistant due to insensitive acetylcholinesterase ( Corbel *et al.*, 2004) and is proposed for use in attractive toxic sugar baits (ATSBs) against mosquitoes ( Khallaayoune *et al.*, 2013). This active ingredient was shown not to be active against *Aedes aegypti* in the absence of an adjuvant by Hoppé *et al.* (2016), whereas the activity was shown in the present study to be enhanced by the addition of RME and it was included in the final short list. 

### Speed of kill

Pyrethroids and other classes of insecticide that have, until recently, been used in vector control are fast acting, and it is upon the particular modes of action of these compounds that the current WHO guidelines for screening compounds for efficacy and cross-resistance are based ( World Health Organisation, 2006). However, there is a growing number of chemistries being repurposed from agricultural and other uses into mosquito vector control, which do not rapidly knock down mosquitoes or kill within 24 hours, but which can still be effective in controlling malaria vector mosquitoes when used in IRS or LLIN products. Notably, reports of trials of clothianidin as an IRS indicate much longer residual efficacy if mortality of exposed mosquitoes is assessed for several days after exposure than if the standard 24-hour endpoint is used ( Agossa *et al.*, 2018; Uragayala *et al.*, 2018). For these compounds, assessing the mortality over a longer period post-exposure and even assessing sub-lethal effects may need to be taken into consideration ( Oxborough *et al.*, 2015b). The focus of this study was to identify chemistries suitable for use in LLINs, where fast-acting compounds give the best personal protection, but future iterations of the testing cascade should perhaps monitor mortality for longer. Sulfoxaflor, spinetoram, fenpyroximate and pyrimidifen were judged to be fast acting and ivermectin, abamectin and metaflumizone particularly slow acting based on topical application. No compound exhibited substantial knock down activity in tarsal contact assays in the absence of RME but, with the addition of RME, the majority of mosquitoes exposed to dinotefuran, fenpyroximate, pyridaben, fenazaquin and tolfenpyrad were affected immediately post-exposure at the breakpoint concentration. The differences in observation between these two bioassays may be due to a difference in the means of exposure. Speed of action is a function of both the mode of action and the physical properties of a compound so that even compounds that act on the nervous system of insects may be slow acting if it takes the compound some time to traverse the cuticle barrier and reach the target site. RME seems to help speed up the movement of some active ingredients across the cuticle, thus speeding up their action and making them effective at lower doses. Several compounds were highly effective based on mortality at 24 hours, but had extremely low knock down rates, which must be taken into consideration when developing new treated net products as exposure to them on a LLIN may not result in any significant blood feeding inhibition. Therefore, when considering possible mixture partners of compounds for use in LLINs, it will be important to consider speed of action and to pair compounds where at least one of them is fast acting. 

In the tarsal contact assay, no great difference was detected between mortality at 24 hours and 48 hours, and in the topical application screen the increase in mortality was not great over this same period for any compound tested. It is possible, however, that longer periods of mortality assessment might have shown a greater efficacy for some of the slower-acting, non-neurotoxic compounds, as has been reported for clothianidin ( Agossa *et al.*, 2018; Uragayala *et al.*, 2018). The high level of knock down and 24-hour mortality seen with clothianidin seems to be at variance with these studies, although the high potency of the compound may mean that the concentrations used in the tarsal contact assay, standard for all compounds in the screen, were high enough to cause a rapid effect which might not be seen at operational doses. The speed of action judged from topical and from tarsal exposure routes were not in complete agreement, an observation which warrants further investigation.

### Additional compounds considered for their potential value

During the course of this study, six additional compounds were considered for inclusion, but not taken through the complete testing cascade (Supplementary Data, included in Raw Data file). Diflumetorim, a mitochondrial complex 1 inhibitor, and acynonapyr, an acaricide with unclassified mode of action, were screened for tarsal activity with RME, but did not reach 80% mortality at the highest concentration tested (125 mg/m ^2^) ( Lees *et al.*, 2019). Pyflubumide, IRAC class 25A, a Beta-Ketonitrile derivative, pyriministrobin, IRAC class 20, and flometoquin, unclassified by IRAC, were screened for tarsal activity both with and without RME but did not reach 80% mortality at the highest concentration tested (125 mg/m ^2^) in either assay ( Lees *et al.*, 2019). Fluralaner (IRAC Class 30), a GABA-gated chloride channel allosteric modulator and, therefore, representing a novel mode of action for public health, is currently under consideration for use as an endectocide ( Miglianico *et al.*, 2018). In this screening study it was effective in tarsal contact assays at the higher concentrations, particularly when mortality was scored at 48 hours, both with and without the presence of an adjuvant ( Lees *et al.*, 2019). Very little knock down was observed at any concentration.

### The shortlisted compounds: 11 repurposed chemistries with potential for use in public health

A total of 11 compounds from eight IRAC classes and oxazosulfyl (which is unclassified by IRAC) were shortlisted at the conclusion of this testing cascade on the basis of their biological efficacy against a pyrethroid susceptible lab colony of *Anopheles gambiae*. Two of these, both from the neonicotinoid IRAC class 4, have now been formulated into products for malaria control: clothianidin is the active ingredient in two IRS products, SumiShield™ 50WG (from Sumitomo Chemical) and Fludora®Fusion (from Bayer Crop Science, in combination with deltamethrin); and dinotefuran is being evaluated in trials of attractive targeted sugar baits ( Khallaayoune *et al.*, 2013). Oxazosulfyl is in development against rice pests by Sumitomo under the trade name Alles™. Indoxacarb (IRAC class 22 Voltage-dependent sodium channel blocker) was developed by DuPont as an oxadiazine pesticide against lepidopterans and has been formulated into a line of commercial pesticidal products by Syngenta: Advion and Arilon. Spinetoram (IRAC class 5) is a derivative of biologically active substances (spinosyns) produced by the soil actinomycete *Saccharopolyspora spinosa*, discovered Dow AgroSciences LLC. and sold as Radiant® SC. Spinetoram affects nicotinic acetylcholine receptors and GABA receptors on postsynaptic membranes in insect nervous systems, thereby causing abnormal neural transmission. Metaflumizone is a novel semicarbazone insecticide (IRAC class 22B) derived chemically from the pyrazoline sodium channel blocker insecticides (SCBIs) discovered at Philips-Duphar in the early 1970s. This compound is a novel sodium channel blocker insecticide, which blocks sodium channels by binding selectively to the slow-inactive state which is characteristic of SCBIs ( Salgado & Hayashi, 2007). Abamectin is a macrocyclic lactone that acts through chloride channel activation. Discovered in 1981, this compound has been shown to be active in contact bioassays against house flies, cockroaches and fire ants, and has been employed in combination with fenpyroximate as a novel durable wall lining ( Malima *et al.*, 2017). Sulfoxaflor is from the sulfoximine class of compounds (IRAC class 4C) which are structurally distinct from neonicotinoids. This compound is marketed by Dow AgroSciences as Isoclast™ Active for use against sap-feeding insect pests. Triflumezopyrim, belongs to the novel class of mesoionic insecticides (IRAC class 4E), binding to the orthosteric site of the nicotinic acetylcholine receptor and, therefore, also distinct from the neoniconoids ( Cordova *et al.*, 2016). Triflumezopyrim is being commercialized by Corteva Agriscience for control of hoppers, including the brown planthopper, *Nilaparvata lugens*, including populations showing resistance to neonicotinoids such as imidacloprid ( Zhang, 2017). Tolfenpyrad and fenpyroximate are classified by IRAC in Group 21A mitochondrial electron transport inhibitors which inhibit the electron transfer system of energy metabolism and respiration in the mitochondria of susceptible insects. Tolfenpyrad has been employed within an ATSB system in studies in Tanzania ( Stewart *et al.*, 2013), and fenpyroximate is a broad spectrum acaricide which has been employed with abamectin as a novel durable wall lining ( Malima *et al.*, 2017).

There are non-entomological parameters to be considered before deciding which of the shortlisted compounds should be progressed for consideration as a component in a vector control product, including physiochemical properties and suitability for use on an LLIN, human safety risk assessment and cost of production. Crucially, the efficacy of these compounds must also be assessed against mosquitoes resistant to pyrethroids and other existing classes of insecticide to check for any pre-existing cross-resistance. Although the modes of action of these shortlisted compounds are novel to mosquito control, cross resistance in field strains may occur through metabolic detoxification which is not related to mode of action ( David *et al.*, 2013) and is the predominant form of resistance in populations of *Anopheles funestus*, where the kdr point mutation is absent ( Tchigossou *et al.*, 2018). Cross resistance, should it be identified, would likely make a compound unsuitable for progression.

## Conclusion

A screening cascade for evaluating the potential of existing insecticidal compounds for repurposing into control tools against malaria vectors has been developed. This was used to screen 40 compounds for intrinsic activity and tarsal contact activity, and to determine relative potency. The inclusion of the adjuvant RME was shown to be critical to avoid eliminating compounds where efficacy could be improved through formulation. A more streamlined testing cascade is suggested, that relies on topical application and determination of discriminating dose in the presence of an adjuvant. This would allow for screening of compounds to progress more quickly, but without losing critical information, although further validation of this streamlined testing cascade is required. There were 11 compounds were identified offering novel modes of action in public health, a critical step in the development of products that can control insecticide resistant populations of Anopheline vectors of malaria. The next key step in progressing these compounds towards product development will be to assess their efficacy against strains of *Anopheles* expressing resistance to existing vector control insecticides, to assess cross resistance risk. Additional information, beyond entomological efficacy, must also be considered when assessing the suitability of these compounds for development into new vector control tools, including safety, cost efficacy, and physicochemical properties relating to product formulation.

## Data availability

Figshare: A comprehensive testing cascade to identify resistance breaking repurposed insecticides for next generation vector control tools: screening a panel of chemistries against a malaria vector - Raw Data. https://doi.org/10.6084/m9.figshare.c.4490246.v1 ( Lees *et al.*, 2019).

This collection contains the following underlying data:

- Raw data Phase 1 Topical Application.xlsx (raw data for topical application of 40 compounds for 60-min knock down and 24- and 48-hour mortality).
- Raw data Phase 2 Tarsal Contact Assay.xlsx (60-min knock down, and 24- and 48-hour mortality observations of mosquitoes exposed to insecticides applied to a glass petri dish).
- Raw data Phase 3 Discriminating Doses.xlsx (24-hour mortality data from CDC bottle bioassay dose response experiments used to calculate LC values for 11 compounds).

Data are available under the terms of the Creative Commons Zero "No rights reserved" data waiver (CC0 1.0 Public domain dedication).

## Author information

Anthony White is a Scientific Advisor to IVCC, Liverpool School of Tropical Medicine, Liverpool, UK.
