# Supplementary material for: A testing cascade to identify repurposed insecticides for next-generation vector control tools: screening a panel of chemistries with novel modes of action against a malaria vector
Source: Gates Open Res. 2019 Jul 10;3:1464. Originally published 2019 May 13. [Version 2] doi: 10.12688/gatesopenres.12957.2 (PMC6556762; doi:10.12688/gatesopenres.12957.2)
Supplement: Peer review history

# Peer review: Reviewer response for version 1

**DOI:** 10.21956/gatesopenres.14063.r27185

**Published:** 2019-06-24

**Authors:** Richard M. Oxborough

**Affiliations:** 1 PMI VectorLink Project, Abt Associates, Rockville, MD, USA

This is a very important study, given the relative lack of insecticides currently registered for malaria control. The methods used were generally appropriate. I agree with the authors that the petri dish bioassays are not necessary & that topical application followed by bottle bioassay is a better testing cascade. 

 Overall, the paper is well-written & I only have a few minor comments for the authors to address. 

- **Title**

I would recommend removing 'comprehensive'. As the authors point out, flupyradifurone was rejected based on the topical screen, but has since been developed as a space spray. 

 I also don't agree with the term 'resistance breaking'. This is a time-sensitive terminology. Bendiocarb could have been considered resistance breaking when first used for IRS, but now that resistance has developed it would no longer be accurate. 

 Suggest title to be "A testing cascade to identify repurposed insecticides for next-generation vector control tools: screening a panel of chemistries against a malaria vector".

- **Introduction (& throughout)**

Long-lasting insecticidal net should be abbreviated as LLIN and not LN. LN is a specific abbreviation used by WHO to denote the 'formulation'. e.g. Sumishield WG, Interceptor LN. However, for general use LLIN should be the abbreviation for long-lasting insecticidal net. 

- **Introduction**

One of the key findings from the paper is the use of RME as an adjuvant which had a huge effect on mortality for several insecticides. It would be great if you can add more background about RME. Has it been used previously in mosquito bioassays? Where did this idea come from? Any additional background info you can provide would be very useful. How did you decide on 100g/m2 RME for Petri dish and 1.5mg/bottle? 

- **Figure 1**

Text related to Phase 1 mentions '>1' means that activity was not observed even when the highest concentration was used. I don't see >1 anywhere in the diagram, only 0.01, 0.1, 1. 

- **Methods (holding period)**

I was initially surprised to see results reported at only 24h, especially considering clothianidin formulations have been held for several days in other papers. However, it was good to see that there is a section in the discussion on speed of kill. I would recommend for future cascade testing to increase the holding period to at least 72h. 

- **Methods (dosages tested)**

It wasn't clear to me why 1% was chosen as the maximum for topical bioassay & 125mg/m2 for petri dish. 

- **Results (pg 8)**

Fenazaquin was rejected due to low topical application but passed tarsal bioassays. I think this is an important finding to flag up in the discussion as there are bound to always be some insecticides that fail 1 type of screening but may still be of use. 

- **Figure 5**

It is not clear why the chemical structures are presented in this paper. There is no discussion of the relevance in the text. Suggest to either add text related to the chemical structures or remove the figure. 

- **Discussion (pg 12)**

"all showed little or no activity in the tarsal contact assay". This ignores Fenazaquin, which failed topical screening but killed 80% in tarsal bioassays.

I confirm that I have read this submission and believe that I have an appropriate level of expertise to confirm that it is of an acceptable scientific standard.

# Response

**Published:** 2019-06-28

**Authors:** Rosemary Lees

**Affiliations:** Liverpool School of Tropical Medicine, UK

The authors are grateful to the Reviewer for some very constructive comments. We have taken on board all comments and adjusted the text and figures accordingly.

The comment regarding the use of the word ‘comprehensive’ in the title is well taken, and indeed we discuss in the text how other modes of exposure may identify compounds which are not active via tarsal or topical contact.  The focus of this study was LLINs, but a fully comprehensive screen could include screening aerosolised compounds, or ingestion in a blood or sugar meal. Regarding the use of the phrase ‘resistance breaking’, we do feel that the testing cascade is designed to identify chemistries that aren’t, to our knowledge, cross-resisted by mechanisms of resistance that have evolved to insecticides that have previously been used, or are currently used, in mosquito vector control. We think that it is generally understood that insecticides considered to be resistance breaking at a given point in time won’t be resistance breaking forever. However, we have altered the wording of the title to focus on the search for new modes of action, which is of course a means of overcoming resistance.

The authors agree that the benefits of including an adjuvant in a screen of this sort is one of the key findings.  The idea for its use arose from another project that IVCC was involved in, and based on the apparent improvement of efficacy when it is included we decided to include it as a step in the testing cascade.  The concentration used for the tarsal contact assay was selected based upon field application rates of RME-herbicide mixes, but this concentration was found to be too high in bottle bioassays where mosquitoes were observed to stick to the coated bottles on some occasions, and so the concentration was reduced. The lower concentrations were validated as giving the same adjuvant effect in tarsal bioassays as the original concentration before use in the bottle bioassays. We have cited all the published information that we are aware of regarding the use of RME as an adjuvant which hopefully now gives sufficient background for this methodology and finding.

The speed of kill is indeed an important issue when considering new chemistries for vector control, and we have edited the text to make sure this is addressed clearly. The focus of this study was to identify compounds suitable for use in non-pyrethroid LLINs where fast acting chemistries are desirable to provide personal protection through quick knock down and kill. But certainly there are ways to employ slower acting chemistries, and longer observation times are relevant for many of the new classes of insecticide.

The inclusion of the chemical structures is for the readers interest and ease of reference, since we have not, as the reviewer highlights, considered their significance or common structures. We have used ISO common names throughout the text, and so we hope that the structures add clarity for those who are interested without detracting too much from the flow of the paper from those who are not.

The concentrations chosen for the topical application and tarsal contact bioassays were designed to identify compounds with activity in a similar range to a permethrin standard, and to judge potency against this compound. Compounds requiring a much higher concentration to achieve efficacy were not of such great interest.

The reviewer is right that Fenazaquin strictly passed the tarsal contact phase of screening where it was rejected based on topical application, seemingly because it was on the edge of efficacy as judged by the criteria of this testing cascade 70% mortality in highest concentration in top app, and 80% mortality at the highest concentration in tarsal (with RME only). It was not judged to be sufficiently potent to be worth pursuing at this stage, but as the reviewer mentions is still may be of interest for some product classes and depending on other characteristics.

We have made the changes to Figure 1 and the abbreviation used for long-lasting insecticidal nets as suggested, and thank the reviewer for highlighting these points.

# Peer review: Reviewer response for version 1

**DOI:** 10.21956/gatesopenres.14063.r27186

**Published:** 2019-06-03

**Authors:** Catherine A. Hill

**Affiliations:** 1 Department of Entomology, Purdue University, West Lafayette, IN, USA

**General Comments:**

- The manuscript of Lees et al., was a pleasure to read. It is extremely well written, there is logical presentation of both the experimental work and the discussion, the experimental approach is valid, the data are well presented, and the conclusions are appropriate.
- The manuscript represents a robust body of work and is significant on several levels: the authors describe a methodical screening approach to analyze registered pesticides representing major insecticide classes recognized by the IRAC, and identify chemistries with intrinsic contact toxicity to adult Anopheles mosquitoes. They identified eleven actives with modes of action distinct from existing mosquito control chemistries. These chemistries have potential for use as resistance breaking products and re-purposing in LNs and possibly IRS. Given the limitations of existing screening regimes and chemical discovery programs, coupled with the urgent need for new mode of action insecticides, the approach is refreshing, and the results of significant practical impact.
- Several features of the study are very attractive. First, the authors describe a primary screen based on adult mosquitoes; this is unique and has potential to accelerate the discovery process for adulticides in particular. Second, their data suggest that the inclusion of an adjuvant in the secondary tarsal assay may improve hit rate and enhance recovery of actives. Lastly, their data suggest that activity detected in an adult topical assay may be indicative of activity in the tarsal screen, and that the insecticide discovery process could be further streamlined by elimination of one of these steps.
- Figure 1 while detailed, is particularly effective as an overview of a complex screening process comprising multiple decision points
- The discussion is lengthy but thorough, addressing all the questions that came to mind for this reviewer regarding such points as further testing on resistant strains and in combination with existing products etc.
- The authors provide rational for use of a DD value that is 3 X the LC _95_ – good – and make clear their case for a further streamlining their screen.

 
**Minor revisions to be made at discretion of authors            **

- Page 12, para 3, line 11; “…this provides…” Plural?
- Figure 2. This reviewer found the figure difficult to view. The juxtaposed red and green segments may be a challenge for some readers, and it is difficult to identify the segment representing compounds rejected for low tarsal activity. The authors might consider to redraw this figure.
- Could Figure 4 perhaps be strengthened by inclusion of compound names? This would make it easier for readers to cross reference between figures and tables.

 
**Further Questions/Considerations**

- The object of the study is a high-throughput primary screen to rapidly identify adulticides. Typically primary screens test in triplicate/quadruplicate at single, low dose. Thinking ahead, do the authors see other ways to increase capacity/speed by revisions to the screen?
- The reviewer wondered if it might be possible to reserve the modified CDC bottle assay tertiary screen for the small number of high priority chemistries likely to move forward in development, but the disconnect between tarsal and bottle assay data, makes it clear that this is not possible. 

I confirm that I have read this submission and believe that I have an appropriate level of expertise to confirm that it is of an acceptable scientific standard.

# Response

**Published:** 2019-06-28

**Authors:** Rosemary Lees

**Affiliations:** Liverpool School of Tropical Medicine, UK

The authors thank the reviewer for the positive review and detailed engagement with our manuscript.  Regarding the minor comments, we appreciate your suggestions and have followed them all in preparing version 2.

Regarding the streamlining of the testing cascade to increase capacity, we did not envisage this protocol as a way to screen large numbers of compounds, for which we would recommend using a larval screen (as mentioned in the Introduction).  Since the aim was to repurpose existing insecticides, a lot of information was available about the compounds aside from biological activity, and based on this a list of only 40 compounds was taken through the testing cascade, a suitably manageable number of compounds for this strain. We have strengthened this point by a sentence added to the Discussion. That being said, the testing cascade could be made more high throughput by using a system such as that of Hoppé et al (2016) using 12 or even 96 well plates to expose adult mosquitoes to more compounds in a single assay, possibly with some level of automation. As we mention in the Discussion, topical application alone might be sufficient to determine biological activity, and along with determining potency in the form of discriminating dose might be sufficient as a testing cascade without other tarsal contact tests.

The authors agree that the more laborious work involved in determining a discriminating dose using the modified CDC bottle bioassay should be kept only for compounds with potential to be developed further. But as the reviewer mentions, and as addressed in the Discussion, the results do not correlate well with the tarsal assay results; in the current testing strategy the bottle bioassay is required to determine relative potency between compounds.

# Peer review: Reviewer response for version 1

**DOI:** 10.21956/gatesopenres.14063.r27184

**Published:** 2019-05-29

**Authors:** Lizette L. Koekemoer

**Affiliations:** 1 Wits Research Institute for Malaria, University of the Witwatersrand, Johannesburg, South Africa

This paper highlights the development of a pipeline - procedure (screening cascade) to evaluate a number of new chemistries against an African malaria vector. The paper is well written, clear and the authors provided sufficient information under methodology section for the reader to follow.

 With increase reports of insecticide resistance to current classes of insecticides, there is an urgent need to expand chemical compound evaluations to identify novel compounds. A standardized procedure will assist in comparison of data between different stakeholders and the paper addresses the need for the development of this screening cascade.

 It would be good for the authors to standardise on acronyms such as LLINs (Long-lasting insecticidal nets) as per WHO format and not LNs. I am in favour of indexing the paper.

I confirm that I have read this submission and believe that I have an appropriate level of expertise to confirm that it is of an acceptable scientific standard.

# Response

**Published:** 2019-06-28

**Authors:** Rosemary Lees

**Affiliations:** Liverpool School of Tropical Medicine, UK

The authors thank the reviewer for a positive review. The comment regarding the WHO standard use of the acronym LLIN is well taken; we have updated this acronym and checked all others for consistency.
